# Botulinum Neurotoxins and Cancer—A Review of the Literature

**DOI:** 10.3390/toxins12010032

**Published:** 2020-01-05

**Authors:** Shivam O. Mittal, Bahman Jabbari

**Affiliations:** 1 Head, Section for Parkinson’s Disease and Movement Disorders, Cleveland Clinic Abu Dhabi, Abu Dhabi 112412, UAE; shivamommittal@gmail.com; 2 Department of Neurology, Yale University School of Medicine, New Haven, CT 06519, USA

**Keywords:** botulinum toxin, botulinum neurotoxin, cancer, cancer cells, neuropathic pain, post-surgical pain, parotid gland, submaxillary gland, gustatory hyperhidrosis, sialocele, parotid fistula

## Abstract

Botulinum neurotoxins (BoNT) possess an analgesic effect through several mechanisms including an inhibition of acetylcholine release from the neuromuscular junction as well as an inhibition of specific pain transmitters and mediators. Animal studies have shown that a peripheral injection of BoNTs impairs the release of major pain transmitters such as substance P, calcitonin gene related peptide (CGRP) and glutamate from peripheral nerve endings as well as peripheral and central neurons (dorsal root ganglia and spinal cord). These effects lead to pain relief via the reduction of peripheral and central sensitization both of which reflect important mechanisms of pain chronicity. This review provides updated information about the effect of botulinum toxin injection on local pain caused by cancer, painful muscle spasms from a remote cancer, and pain at the site of cancer surgery and radiation. The data from the literature suggests that the local injection of BoNTs improves muscle spasms caused by cancerous mass lesions and alleviates the post-operative neuropathic pain at the site of surgery and radiation. It also helps repair the parotid damage (fistula, sialocele) caused by facial surgery and radiation and improves post-parotidectomy gustatory hyperhidrosis. The limited literature that suggests adding botulinum toxins to cell culture slows/halts the growth of certain cancer cells is also reviewed and discussed.

## 1. Introduction

Currently, there are vast indications for the use of botulinum neurotoxins (BoNT) type A and B in clinical medicine. Their specific inhibitory action on cholinergic synapses makes them desirable for the treatment of several hyperkinetic movement disorders as well as symptoms caused by glandular hyperactivity (sialorrhea and hyperhidrosis) and bladder dysfunction [1]. Disease-oriented reviews indicate that these agents are frequently used for the treatment of spasticity in several common disease conditions such as stroke, cerebral palsy, multiple sclerosis, cerebral, and spinal cord injury [2]. The efficacy of BoNT therapy in migraine headaches, predicted by early investigators [3], has been proven via two large, multicenter clinical trials leading to the approval of onabotulinumtoxinA for the treatment of chronic migraine [4]. Animal and human studies have shown that the local injection of botulinum toxins has an analgesic effect and can relieve several forms of neuropathic pain [5,6,7]. The data indicate an analgesic activity for BoNTs in a wide range of pain disorders that include both neuropathic and non-neuropathic pain.

In recent years, several publications have drawn attention to the utility of BoNT injections in cancer-related pain syndromes arising either by direct pressure from a neoplastic mass or from neuropathic pain at the site of cancer surgery or radiation [8]. Aside from pain, BoNT injection into parotid or submaxillary glands has been shown to reduce symptoms such as sialorrhea resulting from gland injury as well as healing surgical complications such as fistula and sialocele [9]. BoNT injections have been reported to relieve gustatory hyperhidrosis resulting from parotid and oral surgery in cancer patients [10]. The limited literature also suggests that adding BoNT to the culture of cancer cell lines slows growth and mitotic activity of certain cancer cells and promotes apoptosis [11]. 

This review is based on a literature search using the search engines of Pub Med, Ovid embrace, and Google Scholar from 1989 to 1 September 2019. The terms botulinum toxin, botulinum neurotoxin, onabotulinumtoxinA, incobotulinumtoxinA, abobotulinumtoxinA, and rimabotulinumtoxinB were crossed with cancer pain, postsurgical cancer pain, post-radiation cancer pain, salivary glands, sialorrhea, gustatory sweating, cancer cells, and cancer cell line. Book chapters that were written over the past 10 years focusing on the subject of botulinum toxin therapy in cancer patients were also reviewed. The inclusion criteria encompassed all articles found via those three afore-mentioned search engines using the above-mentioned search words. Inclusion required that the manuscript’s abstract contain both words cancer (or neoplasm) and botulinum toxin (or botulinum neurotoxin) therapy. Manuscripts that did not have both cancer and botulinum toxin therapy noted in the abstract were excluded. Manuscripts with benign mass lesions were also excluded. 

## 2. Results

**Figure 1:**
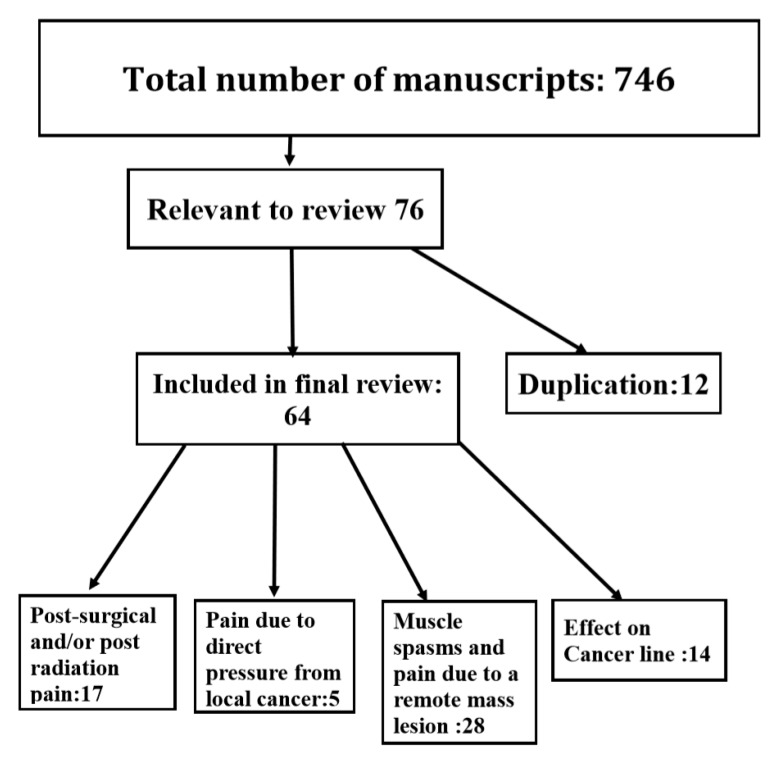
Flow chart of the reviewed manuscripts on cancer and botulinum neurotoxin therapy.

The search identified 746 manuscripts from which 76 were relevant to the subject of botulinum toxins and cancer (see flow chart in Figure 1). After eliminating 12 duplications (due to an overlap between MedLine and Google Scholar), 64 manuscripts remained for final analysis. The collected data can be classified under 3 categories: (1) The role of botulinum toxins in post-radiation and post-surgical cancer pain; (2) the repairing and healing function of BoNT injections upon parotid gland damaged by radiation or surgery; and (3) the effect of botulinum toxins on cancer cell line, cell growth, and apoptosis. 

### 2.1. Botulinum Neurotoxins Therapy for Post-Radiation And/Or Post-Surgical Cancer Pain

**Figure 2:**
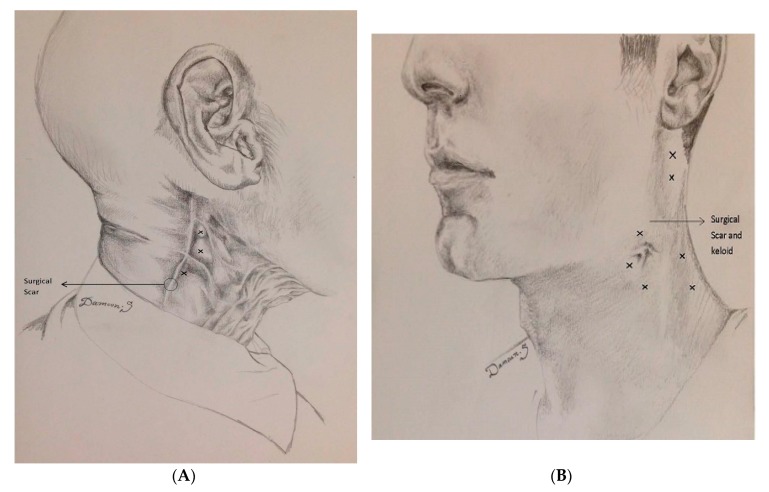
Post-surgical and post-radiation pain treated with BoNT. Example of two patients. From Jabbari B. Botulinum Toxin Treatment in Pain Disorders. Springer, New York 2015. Printed with permission from the publisher. (**A**) A 47-year-old man had undergone right neck dissection and radiotherapy for cancer of the tongue and cervical adenopathy 6 years prior to visiting the Yale clinic. A year following surgery and radiotherapy, severe pain (VAS 9–10, both sharp and deep) developed over the right side of the neck which was mostly felt below the mandible and anterior to the angle of the jaw. Injecting onabotulinumtoxinA into the areas designated by X on the figure, (30, 30, and 20 units) reduced the pain significantly (VAS 1) within a week after injection. He remained responsive and satisfied (assessed by PGIC) receiving injections every 4–6 months over 7 years of follow-up. (**B**) A 48-year old man with squamous cell carcinoma of piriform sinus had supraglottic laryngectomy. Two years following neck dissection and radiotherapy, he developed severe pain (VAS 9) over the left side of the neck. The pain was deep as well as sharp and superficial. Injection of onabotulinumtoxinA, 20 units into each superficial pain region (Xs around the jaw) and 30 units into nearby posteriorly located muscles (splenius and trapezius) designated by X reduced the pain to VAS 0–1 level. The total dose was 200 units. The patient enjoyed pain relief with repeat injections over the 3 years of follow-up. Drawings courtesy of Damoun Safarpour M.D.

This heading includes six prospective clinical trials, four retrospective studies, one double blind placebo-controlled study, and six single case reports (Table 1). A majority of patients had burning and searing pain along the region of fibrosis and keloid formation (neuropathic pain). Some also experienced additional local muscle spasms close to the scarred tissue affecting the neck and shoulder muscles. The most affected muscles were sternocleidomastoid, splenius capitus, trapezius, and levator scapulae. Injections were either subcutaneous (close or at the area of keloid and post-surgical scars, see Figure 2) or both subcutaneous and intramuscular. 

A total of 10 of 11 studies used a standardized scale for pain measurement (Visual Analogue Scale: VAS) which in 9 of 10 demonstrated statistically significant improvement of local pain at 4–8 post-injection weeks compared to baseline (*p* < 0.05) (Table 1). Two studies included patient global impression of change (PGIC) in the evaluation, using a 7-grade scale ranging from “very unsatisfied” to “very satisfied”. In both studies, patients expressed significant satisfaction with the results [18,20]. Four studies used a scale for evaluating quality of life. Three of 4 demonstrated significant improvement of quality of life after BoNT-A (onaA and incoA) injection therapy [14,18,20]. One study demonstrated significant reduction of daily opioid use after BoNT therapy [13]. One prospective study provided long-term follow up of up to 82 months [20]. Side effects consisted mainly of transient pain at the site of injection(s) and minor local bleeding. None of the 229 patients, reported in Table 1, demonstrated any serious side effect following BoNT injections. 

Clinical data from case reports includes six single case reports. One publication reported a 50 year-old man with adenocarcinoma of the soft palate, who following radiotherapy, developed trismus and myokymia of the masseter muscles. Trismus and myokymia improved after injection of 25 units of onaA into each masseter muscle [23]. Two other manuscripts described improvement of central neuropathic pain in association with a mass lesion. One described a 55 year-women who developed severe burning pain and allodynia in the distribution of T1 dermatomes bilaterally following partial resection of an angioma at the C7–C8 region. Subcutaneous injection of onaA at 25 sites into T1 dermatomes (100 units on each sides) resulted in a marked reduction of neuropathic pain and allodynia. This effect was sustained with repeated injections over a follow up period of three years [24]. A similar experience with central pain was reported by Nam et al. [25], in a 62-year old man who had developed severe allodynia and neuropathic pain over the posterior aspect of the left thigh contralateral to a frontal lobe malignant brain tumor. A subcutaneous injection of onaA with a total dose of 100 units at 16 sites substantially improved the patient’s neuropathic pain and allodynia over the affected region. In another patient, radiation of a left submandibular chondrosarcoma resulted in hyperactivity of the spinal accessory nerve and gradual painful hypertrophy of the left trapezius muscle. An injection of 90 units of onaA resulted in a substantial reduction of left shoulder pain and diminished the involuntary myokymic movements of the left trapezius muscle [26]. Boukovalas et al. [27] reported a patient with squamous cell carcinoma of the anterior mandible who, following mandibulectomy, bilateral neck dissection, and radiotherapy, gradually developed pain and tightness of the sternocleidomastoid and platysmal muscles associated with Raynaud phenomenon of the lower face. Injection of botulinum toxin (type and dose not mentioned) into the above-mentioned muscles improved painful muscle tightness and reduced the Raynaud phenomena. Schuler et al. [28] described a 47-year old female who, at the scarred skin site of resected melanoma, developed severe neuropathic pain. Injection of onaA, 50 units in a grid-like pattern (injection sites were 1.5 cm apart), resulted in 50% reduction of pain four weeks after BoNT injection.

The duration of action of BoNT injections for pain relief in the above-mentioned studies was 3–6 months (mean 3.9 month). In most studies, the follow up was short term, not exceeding 6–12 months. In some cases, however, patients were followed-up for years with repeated injections. Two patients described in Figure 1 were followed-up for 3 and 7 years (see figure legend). Nine of 11 studies reported no side effects. One study reported increased pain for a few days at the site of injection in one patient, which was followed by baseline pain improvement [14]. One study reported the occurrence of a diffuse maculo-papular rash in one patient 2–3 days after the botulinum neurotoxin injection after which the rash disappeared over a month [20].

### 2.2. Botulinum Neurotoxins Therapy for Post-Radiation or Postsurgical Damage to Parotid Gland

This category includes six prospective clinical trials, 10 retrospective studies, and 12 single case reports (Table 2). All prospective studies are open label. Botulinum toxin treatment was used for the remedy of post-parotidectomy complications such as gustatory hyperhidrosis (GH), post-parotidectomy sialorrhea, fistula, and sialocele formation. 

The positive information of these studies has been supported by several case reports [45,46,47,48,49,50,51,52,53,54,55,56]. Among these 12 case reports, six described healing of post-parotidectomy fistula and four reported on healing of sialocele. One case reported improvement of gustatory hyperhidrosis and another one reported improvement of post-parotidectomy sialorrhea. A total of 11 studies had used type-A and one had used type B toxin. 

In these 11 studies, no serious side effects were reported. One study reported a patient on anticoagulation in whom a small hematoma developed at the site of injection [34]. One study reported dry mouth as the only side effect [38]. One study cited non-specified, minor issues limited to the site of injection [41]. One study mentioned mild transient weakness of the upper lip in two patients [43] and another study described transient weakness of orbicularis oris muscle in one patient [44]. 

The clinical studies cited above investigating the analgesic effect of BoNTs in patients after surgery or radiation therapy and BoNT’s healing effect on parotid glands injured by surgery or radiation strongly suggest the efficacy of BoNTs in cancer patients affected by surgical and radiation side effects. All three type-A FDA approved BoNTs seem to have analgesic effect in post-surgical and post-radiation pain. In case of parotid injury, at least one study (Table 2) suggests that type B is also effective. Although anecdotal observations have demonstrated safety over 3 to 7 years of treatment (cases presented in Figure 1), the long term safety of BoNT therapy in cancer patients needs to be further investigated through controlled, prospective clinical trials. 

### 2.3. The Effects of Botulinum Neurotoxins Injections on Malignant Tumors and Cancer Cell Line 

This category includes 14 studies. In three studies, investigators injected BoNT into a malignant tumor and demonstrated cellular apoptosis and reduction of tumor size [57,58,59] (Table 3). In another six studies, adding BoNT-A to cancer cell cultures reduced cell growth, induced apoptosis, and inhibited mitosis in various cancer cell lines: Prostate, breast, colon, and pancreatic tumors [60,61,62,63,64,65]. In one study, transfection of insulin secreting cells by BoNT-A reduced insulin secretion, suggesting a potential for treatment of insulinomas [66]. In another study, the addition of BoNT-A to Her2 positive breast cancer cell line increased Herceptin efficacy [67]. In one study, authors reported no effect on prostate tumor growth and LNCaP and PC3 cancer cells after exposure to BoNT [68]. In one study, increased tumor oxygenation after the injection of BoNT-A into hepatic sarcoma and fibrosarcoma suggested that the BoNT injection potentially made these tumors more susceptible to chemotherapy [69]. In another study, the injection of onaA into one side of cancerous human prostate increased apoptosis on the injected side (compare to saline injected into other side) [70]. 

## 3. Discussion

Botulinum neurotoxins exert their analgesic effect through two known mechanisms. The inhibitory effect of the BoNTs upon the release of acetylcholine at the neuromuscular junction is mostly responsible for the relief of pain caused by muscle spasms. In the case of neuropathic pain, it is currently believed that the analgesic effect of botulinum injections predominantly results from inhibition of pain neurotransmitters both at peripheral and at central sensory levels [5,6,71,72]. The peripheral injection of botulinum toxin-A into the muscle or close to peripheral nerve endings reduces the release of calcitonin gene related peptide, a major pain transmitter from trigeminal ganglion [73]. Direct exposure of dorsal root ganglia to botulinum toxin-A significantly reduces the thermal sensitivity in the animal model of thermal pain [74]. In the formalin pain model, injection botulinum toxin B into the rat’s paw reduced substance P release from ipsilateral sensory spinal neurons and prevented spinal sensory neuron activation (c-Fos) which occurred after formalin injection [75]. Injection of botulinum toxins into mice hind paw reduces glutamate release from spinal sensory neurons [76]. Intra-articular injection of botulinum toxin in animal models of pain reduces upregulation of transient receptor potential cation channel subfamily V member 1 (TrpV1), a protein closely associated with pain pathophysiology [77]. A central analgesic function for botulinum toxins has been suggested by studies that have shown the presence of cleaved SNAP-25 in medullary and midbrain sensory regions following the peripheral injection of botulinum toxins [78,79]. Further suggestion for central effects of BoNTs comes from the studies that have demonstrated bilateral improvement of pain sensations after the unilateral injection of botulinum toxin in animal models of diabetic neuropathy and acidic saline injection [80,81]. The analgesic effect of BoNTs results from their direct and indirect effects since patients experience analgesia prior to the muscle relaxation [82]. 

Pain is a common symptom in cancer patients and when present often impairs the patient’s quality of life [83]. Approximately 20–60% of the patients with breast cancer and 30% of the patients with head and neck cancer experience chronic pain localized to the site of radiation or surgery [84]. Post-radiation/surgical pain may be treated with the topical application of a hyaluronic acid, calendula officinalis, trolamine, and lidocaine patch [85,86]. However, sustained relief from pain happens only in 25% of the patients using these remedies [87]. Potent systemic analgesic agents such as opioids provide pain relief in many patients but the development of undesirable side effects including nausea, somnolence, constipation, and addiction complicates their use [88]. Botulinum toxin treatment has two major advantages over these pharmacological remedies. Firstly, the effects of the BoNT-A and B injection lasts 3–6 months. Secondly, the BoNT injection has fewer side effects and is safer when compared to potent analgesic agents. Lack of any serious side effect in the studies cited above supports this statement. 

Gustatory hyperhidrosis (Frey syndrome) can be congenital or acquired. Acquired gustatory hyperhidrosis results from injury to the parotid gland or face as well as conditions such as diabetic autonomic neuropathy. Gustatory hyperhidrosis after parotidectomy results from the aberrant innervation of sweat glands from parasympathetic nerves of the parotid region. Facial sweating during chewing and eating is often a cause of social embarrassment. Gustatory hyperhidrosis (GH) is common after parotidectomy and about half of the patients complained of this symptom after surgery [89]. Botulinum neurotoxins via blocking acetylcholine release at autonomic synapses are highly effective in treatment of autonomic dysfunctions such as sialorrhea and hyperhidrosis [90]. In a meta-analysis of literature on Frey syndrome (multiple etiologies) treated with BoNTs, Xie et al. found the effectiveness of BoNT therapy to be present in 98% of the patients [10]. 

Fistula with sialorrhea and sialocele (entrapped saliva with cyst formation) are two common complications of parotidectomy. Treatment of post-parotidectomy fistula consist of pressure dressing, systemic anticholinergic drugs, suction drain insertion, tympanic neurectomy, and surgery [91]. Overall, the results of the above mentioned surgical and medical strategies in the treatment of parotid fistula is disappointing [92]. Furthermore, side effects of anticholinergic therapy such as memory loss, blurring of vision, dryness of the mouth, and urinary dysfunction are not well tolerated, especially in the elderly. Botulinum toxin injections provides a safe and effective way to suppress sialorrhea and to help heal the fistula. 

## 4. Conclusions

The studies of botulinum toxins in post-surgical and post-radiation pain indicated that the local injection of BoNT improved neuropathic pain and local muscle spasm at/or close to the site of surgery and radiation. The proof of efficacy of botulinum toxin therapy in this form of cancer-related pain, however, awaits the results of blinded and placebo-controlled studies. The same conclusion applies to the use of botulinum neurotoxins in gustatory hyperhidrosis and in the management of post-parotidectomy fistula and sialocele where all open-label studies suggest efficacy. The positive effect of BoNTs on different cancer cell lines and their direct effects upon certain cancerous tumors is encouraging. More studies are necessary to verify these results and if verified to devise a methodology through which BoNT injections can safely be used for the treatment of certain human cancers.

## Figures and Tables

**Table 1:** Published studies on the effect of botulinum neurotoxins (BoNT) on local pain resulting from radiation and/or surgery *.

Authors	Pts Study	Toxin	Dose Units	Treatment	Location of Cancer	Primary Outcome	Result
Van Daele et al., 2002 [12]	6 Retro	OnaA	20–25	Radiation chemotherapy	Head and neck	Pain VAS	Complete pain relief in four of six patients. Significant improvement of quality of life using SF36, EQ-5D scales
Layeeque et al., 2004 [13]	48 Pro	OnaA	100	Mastectomy, assessed for pain after expander placement	Breast	(1) Pain assessed by VAS (2) Narcotic use	Less pain in BoNT group (*p* < 0.00001) Less narcotic use in BoNT group (*p* < 0.0001)
Vasan et al., 2004 [14]	16 Pro	AboA	100 to 320	Surgery	Head and neck	Pain (VAS- days 3 and 4 weeks), global. Quality of life	Significant pain reduction (*p* = 0.05); Quality of life improved (*p* = 0.7)
Wittekindt et al., 2006 [15]	23 Pro	OnaA	60–120 160–240	Radiation; surgery	Head and neck	Pain VAS: 28 weeks	Significant reduction of pain (<0.05)
Hartl et al., 2008 [16]	19 Pro	OnaA AboA	50 250	Chemotherapy; radiation	Head and neck	Pain: (VAS) Function: At 4 weeks	Improved pain (*p* = 0.02) Function (*p* = 0.04)
Stubblefield et al., 2008 [17]	23 Retro	OnaA	25–200	Radiation; surgery	Head, neck, breast	Pain (VAS)	Pain improved in Improved in 85% of patients
Mittal et al., 2012 [18]	7 Retro	OnaA	100	Radiation; surgery	Head, neck, breast	Pain (VAS) PGIC At 4 weeks	VAS: Six of seven patients improved: *p* < 0.05 PGIC: Six of seven, very satisfied QoL: Six of seven improved (*p* < 0.05)
Bach et al., 2012 [19]	9 Pro	OnaA	100–400	Radiation and surgery	Head and neck	Pain (VAS) and FDSNP at 4 weeks	Both pain and FDSNP improved (*p* < 0.01)
Rostami et al., 2014 [20]	12 Pro	IncoA	100	Radiation and surgery	Head neck breast	Pain (VAS) and PGIC at week 6	VAS improved (*p* < 0.05) PGIC: Very satisfied QoL improved in 38% of patients (*p* < 0.05)
De Groef et al. 2018 [21]	50 DBPC	onaA	100	Surgery	Breast	Pain measured by VAS	Pain reduction 60% in the BoNT and 40% in saline group (statistically ns)
Mailly et al., 2019 [22]	16 Retro	incoA aboA	20 40	Radiation and surgery	Head and neck	Pain (VAS)	VAS improved *p* < 0.01

* Case reports are not included. onaA: OnabotulinumtoxinA; aboA: AbobotulinumtoxinA; incoA: IncobotulinumtoxinA; VAS: Visual Analogue Scale; PGIC: Patient Global Impression of Change; FDSNP: Functional Disability Scale for Neck Pain; Pro: Prospective; Retro: Retrospective; DBPC: double-blind, placebo-controlled; QoL: Quality of Life.

**Table 2:** BoNT therapy for post-parotidectomy gustatory hyperhidrosis, fistula, sialocele formation, and for post-parotidectomy sialorrhea.

Authors	Design	Pts #	Clinical Problem	Injection Site	Toxin and Dose	Result
Laskawi et al., 2013 [29]	R	10	Post-parotidectomy fistula	Parotid gland	OnaA 30–50 units	Treated within 6 weeks of surgery: Fistulas healed in 9 of 10 patients
Marchese-Ragona et al., 2006 [30]	R	3	Post-parotidectomy fistula	Parotid gland	OnaA 15–20 units	Complete healing of fistula with follow ups 12,18, and 14 months
Nolte et al., 2004 [31]	P	20	Gustatory sweating after parotidectomy	Facial skin	OnaA 3 units/cm	Complete loss of sweating for 12 months
Kuttner et al., 2001 [32]	R	8	GH after parotidectomy	Face	BoNT-A 0.5 units/cm	Stopped facial sweating within one week
Vargas et al., 2000 [33]	P	4	Post-parotidectomy sialocele-pain	Parotid gland	OnaA 30–50 units	Total resolution in 4 weeks in all patients
Steffen et al., 2014 [34]	R	25	Head and neck cancer FHS: (19) Fistula (6)	Parotid gland	OnaA and incoA: Par: 30 U SM: 20 U	FHS: 11 of 19 improved. Fistula: 4 of 6 improved
Machese et al., 2008 [35]	R	8	Head and neck cancer sialorrhea: 6, fistula: 1, and sialocele: 1	Parotid gland	AboA: 100 U	Fistulas healed. Sialorrhea stopped
Eckardt et al., 2003 [36]	R	33	GH after parotidectomy	Face	OnaA 16 to 80 units	Facial sweating disappeared within a week after injections
Cantarella and Barbieri [37]	R	7	GH after parotidectomy	Face	RimaB 2200 units	Cessation of sweating in 6 of 7 patients 4 weeks after injection
Matos Dias et al., 2008 [38]	R	10	GH after parotidectomy	Face	Ona-A 38 units	Sweating stopped
Hatrl et al., 2008 [39]	R	7	GH after parotidectomy	Face	BoNT-A	Sweating and quality of life improved
Pomprasit et al., 2007 [40]	P	9	GH after Parotidectomy	Face	Ona-A 10.6 units	Sweating stopped in 5 and reduced in 4
Cavalot et al., 2000 [41]	P	40	GH after parotidectomy	Face	Ona-A, 2.5/cm^2^	100% response in severe group, 72% response in moderate group
Von Lindern et al., 2000 [42]	R	7	GH after parotidectomy	Face	Ona-A	Sweating stopped after BoNT injection
Laccourreye et al., 1998 [43]	P	14	GH after parotidectomy	Face	Ona-A	All showed total cessation of sweating
Bjerkhoel et al., 1997 [44]	P	15	GH after parotidectomy	Face	Ona-A	Total cessation of facial sweating in 13 patients

Case reports are not included for salivary gland problems related to cancer surgery or cancer irradiation. R: Retrospective; P: Prospective; onaA: OnabotulinumtoxinA; incoA: IncobotulinumtoxinA; aboA: AbobotulinumtoxinA; FHS: Functional hypersalivation; Par: Parotid, SM: Submandibular.

**Table 3:** In vivo and in vitro effects of BoNT injection on malignant tumors and cancer cell lines.

Authors	Study Type	Type of Cells or Tissue	Study Design	Results
Vezdrevanis 2011 [57]	In vivo	Prostatic cancer	Injected BoNT into prostate	Tumor size reduction
Ulloa et al., 2015 [58]	In vivo	Glioblastoma cells	Cells with or without transfection by BoNT-C1 injected into mice striatum	By BoNT-C1 blocks the growth of Glioblastoma cells via blocking Syntaxin1
He et al., 2016 [59]	In vivo	Mice with pancreatic tumor	Injected onaA or saline into tumor	Reduced tumor size; increased apoptosis
Karsenty et al., 2009 [60]	In vitro	Prostate LNCaP and PC-3 cell lines	LNCaP and PC-3 cell lines were exposed to onaA	OnaA inhibited LNCasP cell proliferation; had no effect on PC-3 cell
Nam et al., 2012 [61]	In vitro	Breast and colorectal cancer	PLC-γl-transformed cells were exposed to BoNT-A (difficile)	Caused apoptosis and mitotic inhibition
Proietti et al., 2012 [62]	In vitro	Prostate LNCaP and PC-3 cell lines	Prostate cancer cell lines were exposed to incoA	Tumor cell growth slowed down probably due to toxin effect on SV2 receptors
Bandala et al., 2013 [63]	In vitro	Breast T47D cancer cells	Breast T47D cancer cells were exposed to diverse dilutions of BoNT	BoNT via caspase 3, slow down the growth of T47d cells and caused apoptosis
Bandala et al., 2015 [64]	In vitro	Breast cancer cell line	Added BoNT-A to breast cancer cell line	BoNT-A diminished SV2 protein on the surface of breast cancer cells
Rust et al., 2016 [65]	In vitro	Human neuroblastoma cells	Added BoNT-C to human neuroblastoma cell culture	Apoptosis of neuroblastoma cells
Huang et al., 1998 [66]	Invitro	Insulin secreting HIT-T15 cells	Insulin secreting cells were transfected by BoNT-A	Marked reduction of insulin secretion- potential to treat insulinoma
Hajighasemlou et al., 2015 [67]	In vitro	Her2 positive breast cancer cell line	Assessed the effect of BoNT-A on Her2 positive cells responsive to Herceptin	Herceptin efficacy significantly improved
Cheng et al., 2013 [68]	In vitro and in vivo	Prostate cancer cell line in Mice	LNCaP and PC3 cancer cells were exposed to 1 to 10 units of onaA	No effect on tumor growth in LNCaP and PC3 cancer cells
Ansiaux et al., 2006 [69]	In vivo	Fibrosarcoma, hepatosarcoma	BoNT-A injected into the tumor	Increased oxygenation of the tumor and made it more susceptible to chemo and radiotherapy
Coarfa et al., 2017 [70]	In vivo	Prostate of 250 nude mice Four human cancerous prostates	Effect of onaA versus saline injection into cancer cells implanted into rodent’s prostate Assessed the effect of onaA versus saline injection in cancerous prostate before prostatectomy	Increased apoptosis; slowed cancer progression Increased apoptosis in ona-A injected side of prostate

OnaA: OnabotulinumtoxinA (Botox). IncoA: IncobotulinumtoxinA (Xeomin).
